# Supplementary material for: In Situ Growth of PbS/PbI2 Heterojunction and Its Photoelectric Properties
Source: Nanomaterials (Basel). 2022 Feb 18;12(4):681. doi: 10.3390/nano12040681 (PMC8879748; doi:10.3390/nano12040681)
Supplement: Supplementary file 1 [file nanomaterials-12-00681-s001.zip › nanomaterials-1577191-supplementary.pdf]

Supplementary Materials

In Situ Growth of PbS/PbI_2 Heterojunction and Its Photoelectric Properties

Shangxun Yang [1], Jun Han [2,*], Jin Zhang [2], Yingxiu Kong [2] and Huan Liu [2]

1 School of Weapons Science and Technology; Xi'an Technological University, Xi' an 710032, China; ysx070619@163.com
2 School of Optoelectronic Engineering, Xi'an Technological University, Xi' an 710032, China; j.zhang@xatu.edu.cn (J.Z.); kongyingxiu@xatu.edu.cn (Y.K.); liuhuan@xatu.edu.cn (H.L.)
* Correspondence: hanjun513@xatu.edu.cn

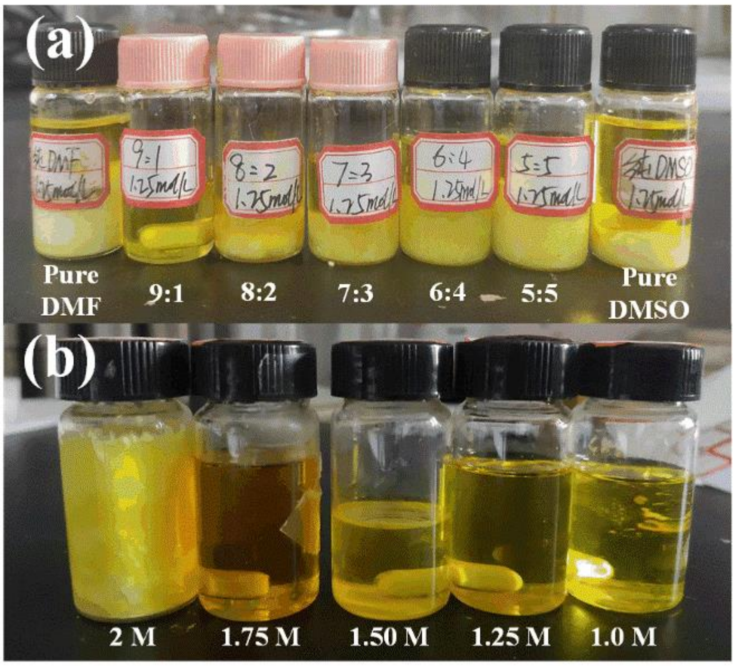

Figure S1. (**a**) Solubility of 1.25 M PbI_2 in mixed solvents with different ratios of DMF to DMSO after ageing for 48 h; (**b**) Solubility of PbI_2 with various concentrations when the ratio of DMF to DMSO is 9:1.

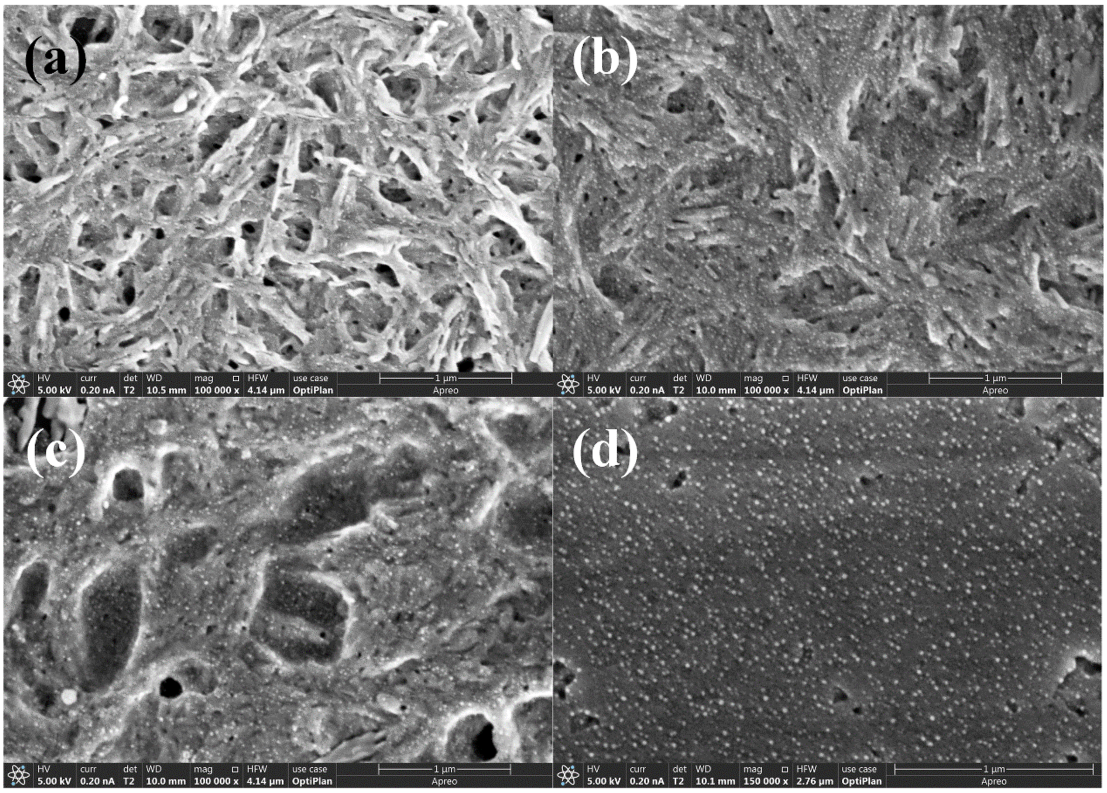

Figure S2. SEM images of PbI_2 thin film prepared with PbI_2 solutions with various concentrations (**a**) 1M; (**b**) 1.25 M; (**c**) 1.50 M; and (**d**) 1.75 M.

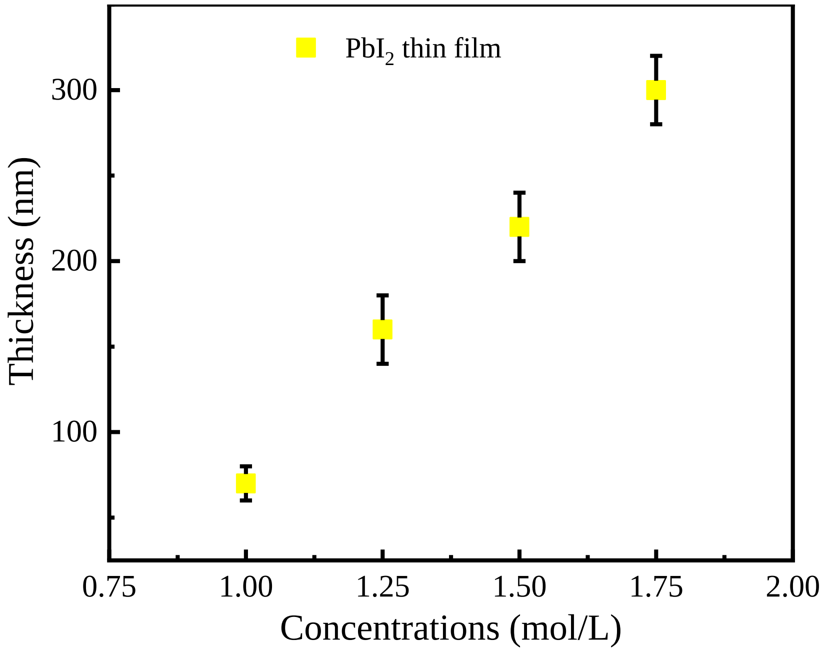

Figure S3. Relationship between the concentration of PbI_2 solution and the thickness of PbI_2 thin film.

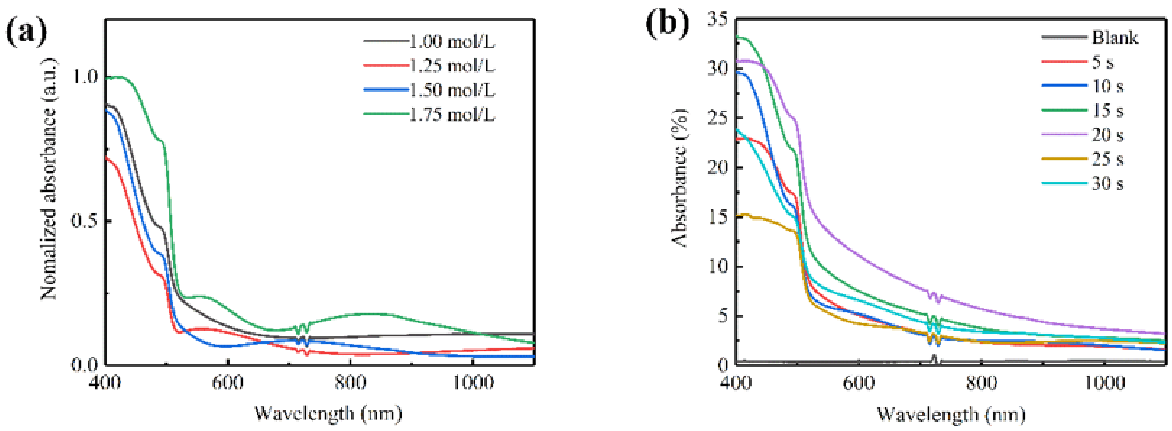

Figure S4. (**a**) Normalized absorbance of PbI_2 thin film prepared with PbI_2 solutions with various concentrations; (**b**) Absorbance of PbI_2/PbS heterojunction at different growing times.

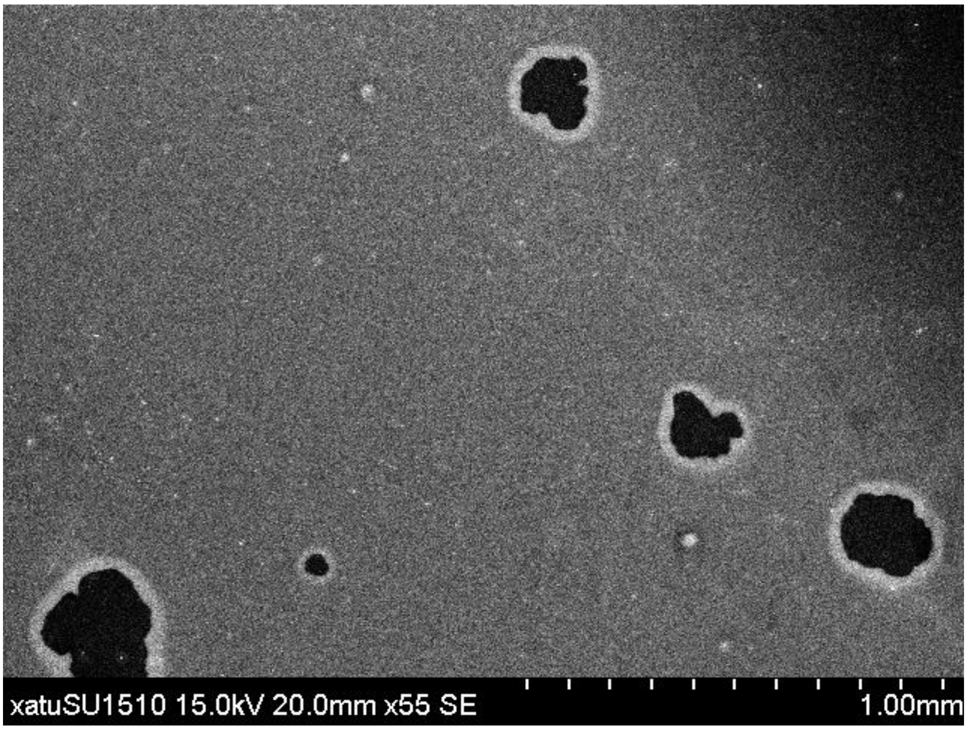

Figure S5. SEM image of PbI_2/PbS heterojunction after growing for 30 s.
